# ZCCHC17 Served as a Predictive Biomarker for Prognosis and Immunotherapy in Hepatocellular Carcinoma

**DOI:** 10.3389/fonc.2021.799566

**Published:** 2022-01-06

**Authors:** Fahui Liu, Jiadong Liang, Puze Long, Lilan Zhu, Wanyun Hou, Xueming Wu, Chunying Luo

**Affiliations:** ^1^ Department of Pathology, Affiliated Hospital of Youjiang Medical University for Nationalities, Baise, China; ^2^ Undergraduate Clinical Medicine, Youjiang Medical University for Nationalities, Baise, China; ^3^ Department of Cell Biology, Medical College of Guangxi University, Nanning, China

**Keywords:** ZCCHC17, HCC, diagnosis, prognosis, immunotherapy

## Abstract

Hepatocellular carcinoma (HCC) is one of the common malignant tumors. The prognosis and five-year survival rate of HCC are not promising due to tumor recurrence and metastasis. Exploring markers that contribute to the early diagnosis of HCC, markers for prognostic evaluation of HCC patients, and effective targets for treating HCC patients are in the spotlight of HCC therapy. Zinc Finger CCHC-Type Containing 17 (ZCCHC17) encodes the RNA binding protein ZCCHC17, but its role in HCC is still unclear. Here, 90 paraffin-embedded specimens combined with bioinformatics were used to comprehensively clarify the value of ZCCHC17 in the diagnosis and prognosis of HCC and its potential functions. Paraffin-embedded specimens were used to assess ZCCHC17 protein expression and its correlation with prognosis in 90 HCC patients. the public data sets of HCC patients from TCGA, ICG, and GEO databases were also used for further analysis. It was found that protein and mRNA levels of ZCCHC17 in HCC tissues were significantly higher than those in normal tissues. The abnormally high expression may be related to the abnormal DNA methylation of ZCCHC17 in tumor tissues. The high expression of ZCCHC17 is related to AFP, histologic grade, tumor status, vascular invasion, and pathological stage. Multi-data set analysis showed that patients with high ZCCHC17 expression had a worse prognosis, and multivariate cox regression analysis showed an independent prognostic significance of ZCCHC17. The results of functional analysis, including Gene Ontology (GO), Kyoto Encyclopedia of Genes and Genomes (KEGG) and Gene Set Enrichment Analysis (GSEA), indicate that ZCCHC17 is mainly involved in immune regulation. Subsequently, further single-sample gene set enrichment analysis (ssGSEA) showed that the expression of ZCCHC17 was related to the infiltration of immune cells. Importantly, we also analyzed the relationship between ZCCHC17 and immune checkpoint genes, tumor mutation burden (TMB), microsatellite instability (MSI) and TP53 status in HCC patients and evaluated the role of ZCCHC17 in cancer immunotherapy. In summary, ZCCHC17 is a novel marker for the diagnosis and prognostic evaluation of HCC. Concurrently, it regulates immune cells in the tumor microenvironment (TME) of HCC patients, which has a specific reference value for the immunotherapy of HCC.

## Introduction

HCC is a common malignancy of liver cancer. Worldwide, there were 905667 new cases of HCC in 2020, accounting for 4.7% of new cancer cases in the whole year. It is the fourth leading cause of cancer death globally, and the annual mortality rate is as high as 8.3% (1). The early symptoms of most patients are usually not evident, and they have reached the middle-to-late stage when coming to medical attention. Moreover, due to tumor size and location, recurrence, and metastasis, it is difficult for many patients to undergo surgery. The overall survival after treatment is still poor. In recent years, although sorafenib has somewhat improved survival in patients with advanced HCC (2), and Nivolumab and Pembrolizumab have shown positive results in recent immunotherapy clinical trials (3, 4). However, HCC still has a dismal prognosis. Therefore, it is of great significance to explore the pathogenesis of HCC and identify new diagnostic markers and therapeutic targets.

ZCCHC17 is one of the genes encoding RNA binding proteins(RBP) (5). As an essential participant in post-transcriptional modification, RBPs often bind to RNA to form ribonucleoprotein complex (RNP). Defects in RNP structure or function can lead to disease, including tumor formation. Factors that cause abnormal function of RBPs in tumors include genomic changes (6), transcriptional and post-transcriptional modification regulation (7) and post-translational modification (8). RBPs can affect the occurrence and development of tumors by regulating the maturation, translation, localization and stability of RNA and binding DNA (9). Because of the vital role of RBP in post-transcriptional modification, even minor changes can have significant effects. The abnormal function of RBP can often affect multiple tumor signature features, such as tumor proliferation, tumor metastasis, tumor death resistance, tumor metabolic disorder, tumor immune escape, tumor genome instability (10).

In view of the close relationship between RPBs and tumorigenesis, there is insufficient evidence for the role and clinical significance of ZCCHC17 in the diagnosis, disease progression, and prognosis of HCC. In this study, the expression of ZCCHC17 in HCC tissues was studied by collecting clinical samples and bioinformatics methods. The correlation or role of ZCCHC17 in the occurrence, development and prognosis of HCC was analyzed by bioinformatics. To lay an experimental and theoretical basis for further investigation of the mechanism of ZCCHC17 in the occurrence and development of HCC and its influence on the treatment of HCC, and to provide some valuable insights and therapeutic strategies for the treatment in HCC. Importantly, ZCCHC17 appears to affect the prognosis of HCC patients partly through regulating immune cells infiltration. These findings highlight the significant role of ZCCHC17 in HCC and suggest that ZCCHC17 may play an essential role in the regulation of immune cells infiltration in HCC and in guiding the treatment of HCC patients.

## Materials and Methods

### Data Collection of HCC Patients

RNAseq data (level 3 HTSeq-FPKM) of 374 hepatocellular carcinoma patients was downloaded from the TCGA (https://portal.gdc.cancer.gov/) LIHC (hepatocellular carcinoma) project. The formatted RNAseq data were converted into TPM (transcripts per million reads) format for subsequent analysis. The TCGA methylation data evaluated by the Infinium 450K array was obtained from the Xena Public Data Center (https://xena.ucsc.edu/). The RNAseq data in TPM format of TCGA and GTEx processed uniformly by the Toil process (11) is downloaded from UCSC XENA (https://xenabrowser.net/datapages/). 231 HCC patients with complete follow-up data and RNA sequencing data were downloaded from ICGC. Gene expression profiling data sets (GSE54236, GSE84005) were obtained from the GEO database (https://www.ncbi.nlm.nih.gov/gds). A total of 4 sets of data downloaded from public databases were used to study the expression differences of ZCCHC17. The data of HCC patients from TCGA and ICGC with survival data were used for further survival analysis. In addition. In order to further prove the reliability of the experiment, we collected the paraffin-embedded tissues of 90 HCC patients with complete follow-up information from the Department of Pathology, Affiliated Hospital of Youjiang Medical University for Nationalities as a validation cohort. This study was reviewed and approved by the Ethics Committee of the Affiliated Hospital of Youjiang Medical University for Nationalities (YYFY-LL-2021-31), and all patients agreed with written informed consent. The criteria for inclusion were as follows: all of the HCC patients were treated for the first time, and none of the patients received radiotherapy, chemotherapy, or targeted anticancer drug treatment before surgery. The primary lesion was hepatocellular carcinoma, and the pathological type was hepatocellular carcinoma. Exclusion criteria included patients receiving chemotherapy or radiation before surgery or patients diagnosed with multiple neoplasms, cholangiocarcinoma, or metastatic HCC.

### Immunohistochemistry

The collected paraffin-embedded tissues were made into pathological sections with a diameter of 3 to 5 microns. The pathological sections were processed through the following workflow: sliced, dewaxed, antigen repaired (EDTA PH=9.0), and blocked. Slides were incubated in primary antibody (ZCCHC17, Invitrogen, #PA5-103989, 1:1000 Dilution) overnight at 4°C. Secondary antibody incubation(MaxVision, KIT-5010), DAB staining, hematoxylin staining. After dehydration, the sections were sealed with neutral gum. The image on the sections is collected using the tissue slice digital scanner and imaging system, and the image analysis system automatically reads the tissue measurement data. Staining was scored as follows: 0: negative or no coloring; 1: weak positive light yellow; 2: medium positive brown; 3: intense positive brown. Then the positive area of weak, medium and intense in the measuring area is analyzed and calculated separately. The integrated optical density (IOD) in the positive area and the size of the positive area is measured. The mean density value (IOD/positive area) indicated the expression abundance of ZCCHC17 protein.

### Bioinformatics Analysis

The level of methylation for each CpG site is expressed as a Beta value. Beta values ​​ranged from 0 (unmethylated) to 1 (fully methylated). The protein data file that interacts with ZCCHC17 is downloaded in the Compartmentalized Protein-Protein Interaction Database | v2.1.1 (Http://comppi.linkgroup.hu) (12). The functions and pathway analysis of proteins that interact with ZCCHC17 for GO and KEGG were performed using the “clusterProfiler” package (13). The correlation between ZCCHC17 and HCC patients’ prognosis was analyzed using Kaplan-Meier survival analysis and Cox regression analysis. We use the ssGSEA (14) (single-sample gene set enrichment analysis) algorithm to quantify the relative abundance of 24 immune cell infiltrations in the tumor microenvironment of HCC patients. The enrichment score calculated by ssGSEA analysis represents the relative abundance of infiltrating cells in each tumor microenvironment in each sample. Potential ICB response for ZCCHC17 patients in high and low groups was predicted with the TIDE algorithm (15).

### Statistical Analysis

Clinical information was obtained from TCGA and was analyzed by R-4.0.2 and SPSS 24.0. For comparisons of two groups, paired or unpaired t-tests were used for normally distributed variables; otherwise, Wilcoxon signed-rank test was used. For correlations between two variables, Spearman’s correlation test was used. The differences in patient survival with different groups were analyzed using Kaplan-Meier analysis and uni- and multivariate analyses. For all Statistical analyses, A P-value < 0.05 was considered statistically significant in this study.

## Results

### Expression of ZCCH17 in HCC and Diagnostic Efficacy for HCC

**Figure 1:**
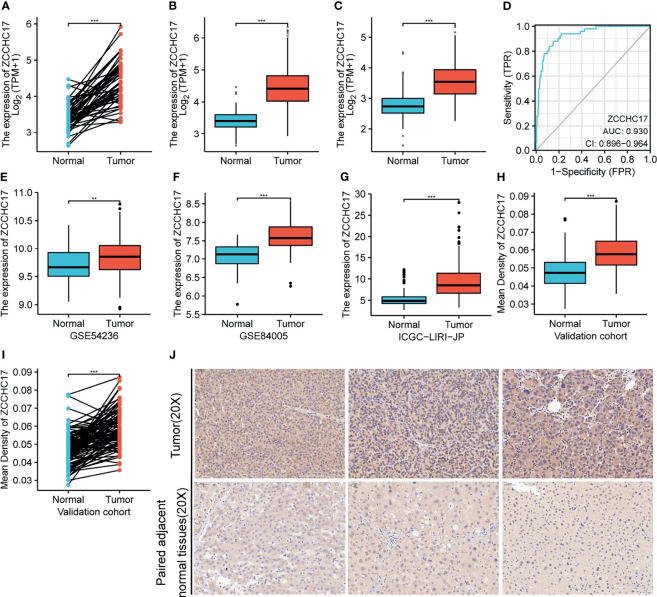
Expression of ZCCHC17 in HCC and diagnostic efficacy for HCC. **(A, B)** ZCCHC17 was highly expressed in HCC in TCGA (P <0.001); **(C)** High expression of ZCCHC17 in HCC from TCGA compared with normal tissues from GTEX and TCGA (P <0.001); **(D)** Diagnostic capability of ZCCHC17 in HCC; **(E)** High expression of ZCCHC17 in HCC in GSE54236 (P <0.01); **(F)** High expression of ZCCHC17 in HCC in GSE84005 (P <0.001); **(G)** High expression of ZCCHC17 in HCC in ICGC; **(H–J)** ZCCHC17 proteins were highly expressed in HCC compared with adjacent normal tissues (20X) (P <0.001). **P < 0.01; ***P < 0.001.

The results showed that the ZCCHC17 expression in the tumor was higher than normal, and the difference between the two groups was 0.935 (0.755-1.114). The difference was statistically significant(t = 10.470, P < 0.001) (**Figure 1A**). The analysis of TCGA unpaired samples showed that the expression level of ZCCHC17 in the tumor was significantly higher than that of normal, and the difference was statistically significant (P <0.001) (**Figure 1B**). In addition, to further determine the accuracy of the results, we added normal samples from GTEx. The comparison results still show that the expression level of ZCCHC17 in the tumor is significantly higher than that of normal (P <0.001) (**Figure 1C**). In the diagnostic value of ZCCHC17, ZCCHC17 showed a high accuracy of tumor prediction in HCC.(AUC = 0.930, CI = 0.896-0.964) (**Figure 1D**). At the same time, we downloaded the expression profile data of HCC patients from GSE54236, GSE84005, and ICGC to validate the expression difference of ZCCHC17 between tumor and non-tumor samples. The results showed that the expression level of ZCCHC17 in the tumor was significantly higher than that in normal (P<0.001) (**Figures 1E–G**). At the same time, through immunohistochemistry experiments of clinical samples. The independent sample T-test showed that the ZCCHC17 in the tumor was higher than normal, and the difference between the two groups was 0.011 (0.008-0.014). The difference was statistically significant (t = 7.683, P < 0.001) (**Figure 1H**). The paired sample T-test showed that the ZCCHC17 in the tumor was higher than that in normal, and the difference between the two groups was 0.011 (0.009-0.014). The difference was statistically significant (t = 9.299, P < 0.001) (**Figure 1I**). It can also be observed that the staining intensity of ZCCHC17 in HCC is also significantly higher than that of paired adjacent normal tissues (20X) (**Figure 1J**). These results indicate that ZCCHC17 also has significant differences in protein expression levels. Ns, P≥0.05; *, P<0.05; **, P<0.01; ***, P<0.001

### DNA Methylation Is Involved in Mediating Changes in ZCCHC17 Expression

**Figure 2:**
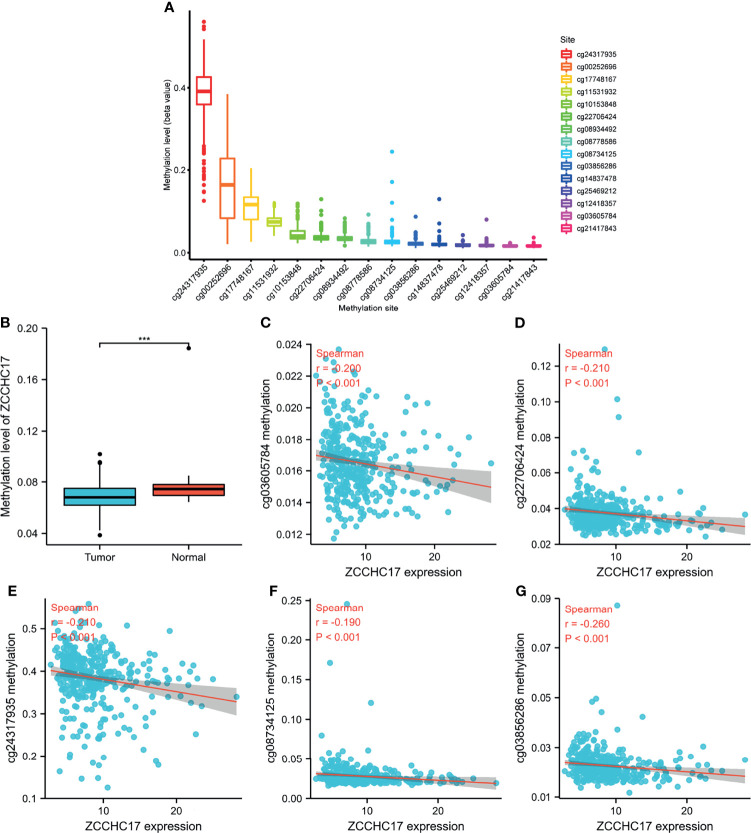
ZCCHC17 methylation levels in HCC from TCGA. **(A)** The methylation level of 15 methylation sites for ZCCHC17; **(B)** Difference in ZCCHC17 methylation levels between normal and tumor tissues; **(C–G)** Correlation of ZCCHC17 methylation level with the expression level of ZCCHC17. ***P < 0.001.

As shown in Figure A, ZCCHC17 has 15 methylation sites, of which cg24317935 has the highest methylation level, and cg21417843 has the lowest methylation level (**Figure 2A**). Then we analyzed the difference in methylation level between the tumor and the normal samples. The results showed that the methylation level in the normal group was significantly higher than that in the tumor group (P <0.001) (**Figure 2B**). Then we analyzed 15 methylation sites. The correlation between the methylation and the expression level of ZCCHC17 showed that cg03605784 (r=-0.200, P <0.001), cg03856286(r=-0.260, P <0.001), cg08734125 (r=-0.190, P <0.001), cg22706424 (r=-0.210, P <0.001), cg24317935 (r=0.-210, P <0.001) was significantly negatively correlated with the expression of ZCCHC17 (**Figures 2C–G**). Ns, P≥0.05; *, P<0.05; **, P<0.01; ***, P<0.001

### The Association Between ZCCHC17 Expression and Clinicopathologic Features

**Figure 3:**
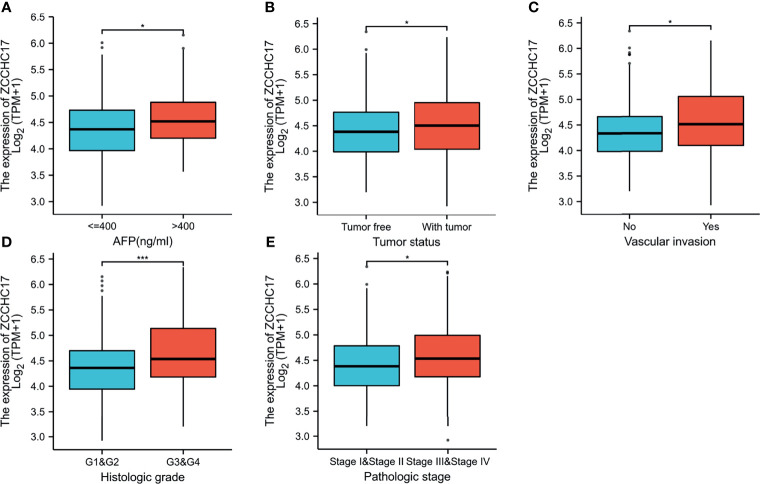
Expression differences of ZCCHC17 in different subgroups in TCGA. **(A)** ZCCHC17 differentially expressed in different levels of AFP (P <0.05); **(B)** ZCCHC17 differentially expressed in different tumor status (P <0.05); **(C)** ZCCHC17 differentially expressed in different groups of vascular invasion(P<0.05); **(D)** ZCCHC17 differentially expressed in different histologic grade(P<0.001); **(E)** ZCCHC17 differentially expressed in different groups of the pathological stage (P<0.05). *P < 0.05; ***P < 0.001.

The characteristics of 374 HCC patients from TCGA, including T stage, N stage, M stage, Pathologic stage, Tumor status, Gender, Age, Histologic grade, AFP, Vascular invasion, were collected and shown in **Table 1**. In order to further understand the clinical significance of ZCCHC17 in HCC, we compared the clinicopathological relationship between ZCCHC17 expression and different subgroups. The results showed significant differences in the expression of ZCCHC17 in different subgroups of AFP, histologic grade, tumor status, vascular invasion and pathological stage (**Figures 3A–E**). Ns, P≥0.05; *, P<0.05; **, P<0.01; ***, P<0.001

**Table 1:** Detailed clinical information for all HCC patients from TCGA.

Characteristic	Low Expression of ZCCHC17	High Expression of ZCCHC17	p
n	187	187	
**T stage**, n (%)			**0.021**
T1	105 (28.3%)	78 (21%)	
T2	38 (10.2%)	57 (15.4%)	
T3	39 (10.5%)	41 (11.1%)	
T4	4 (1.1%)	9 (2.4%)	
**N stage**, n (%)			0.059
N0	130 (50.4%)	124 (48.1%)	
N1	0 (0%)	4 (1.6%)	
**M stage**, n (%)			1.000
M0	130 (47.8%)	138 (50.7%)	
M1	2 (0.7%)	2 (0.7%)	
**Pathologic stage**, n (%)			**0.032**
Stage I	101 (28.9%)	72 (20.6%)	
Stage II	37 (10.6%)	50 (14.3%)	
Stage III	37 (10.6%)	48 (13.7%)	
Stage IV	2 (0.6%)	3 (0.9%)	
**Tumor status**, n (%)			0.305
Tumor free	106 (29.9%)	96 (27%)	
With tumor	71 (20%)	82 (23.1%)	
**Gender**, n (%)			0.658
Female	58 (15.5%)	63 (16.8%)	
Male	129 (34.5%)	124 (33.2%)	
**Age**, n (%)			0.277
<=60	83 (22.3%)	94 (25.2%)	
>60	104 (27.9%)	92 (24.7%)	
**Histologic grade**, n (%)			**0.032**
G1	32 (8.7%)	23 (6.2%)	
G2	95 (25.7%)	83 (22.5%)	
G3	56 (15.2%)	68 (18.4%)	
G4	2 (0.5%)	10 (2.7%)	
**AFP(ng/ml)**, n (%)			0.336
<=400	116 (41.4%)	99 (35.4%)	
>400	30 (10.7%)	35 (12.5%)	
**Vascular invasion**, n (%)			0.086
No	117 (36.8%)	91 (28.6%)	
Yes	50 (15.7%)	60 (18.9%)	

The bold values represent different pathological features. The P value in bold indicates that the P value is statistically significant.

### The Relationships Between ZCCHC17 Expression and Survival in HCC

**Figure 4:**
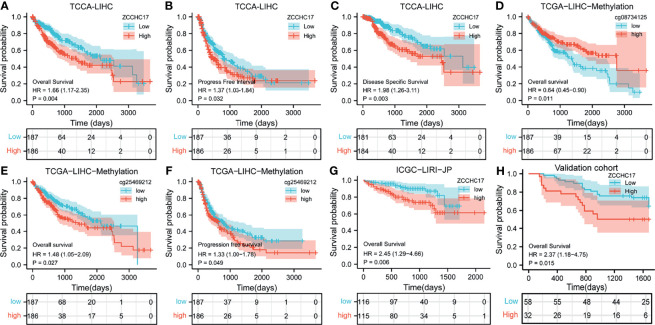
Impact of ZCCHC17 on survival in HCC patients from different cohorts. **(A–C)** The expression of ZCCHC17 was significantly related to patients’ OS (P= 0.004), PFS (P=0.0032) and DSS (P=0.003) in TCGA; **(D)** High methylation level of cg08734125 is relevant to the patient’s better OS (P=0.011) in TCGA; **(E, F)** Low methylation level of cg25469212 is relevant to the patient’s worse OS(P=0.027) and PFS (P= 0.049) in TCGA; **(G)** The poor OS correlated with high ZCCHC17 expression in ICGC(P=0.006); **(H)** High expression of ZCCHC17 indicates a poor OS in the validation cohort. (P= 0.0015).

Owing to the expression level of ZCCHC17 is closely related to the progression and malignancy of HCC, we then tested the prognostic value of ZCCHC17. The results showed that the expression of ZCCHC17 was significantly related to patients’ overall survival (OS), progression-free survival (PFS) and disease-specific survival (DSS) (**Figures 4A–C**). In order to explore the relationship between different methylation sites of ZCCHC17 and prognosis in HCC, we identified the relationship between the 15 methylation sites of ZCCHC17 and the OS, PFS of HCC patients. According to the median of the methylation value, we divided the patients into high and low groups. The results showed that the high methylation level of the methylation site cg08734125 is relevant to the patient’s better OS (P=0.011), and the high methylation level of cg25469212 is related to the poor patient OS (P=0.021). In addition, the high methylation level of cg25469212 was associated with worse PFS in patients (P=0.049) (**Figures 4D–F**). In addition, data from ICGC and validation cohort also proved that increased expression of ZCCHC17 was significantly associated with worse OS (**Figures 4G, H**). Univariate Cox regression analysis showed that Stage III and Stage IV, With tumor, and the expression level of ZCCHC17 was a risk factor for the prognosis of HCC patients. Further multivariate Cox regression analysis showed that Stage III and Stage IV, With tumor, The expression level of ZCCHC17 is an independent risk factor affecting the prognosis of HCC patients (**Table 2**). In summary, the above results suggest that ZCCHC17 plays a vital role in the prognostic evaluation of HCC patients.

**Table 2:** Univariate and multivariate Cox regression analyses.

Characteristics	Total (N)	Univariate Analysis		Multivariate Analysis	
		Hazard ratio (95% CI)	P-value	Hazard ratio (95% CI)	P-value
Pathologic stage	349				
Stage I & Stage II	260	Reference			
Stage III & Stage IV	90	2.504 (1.727-3.631)	<0.001	2.049 (1.375-3.054)	<0.001
Tumor status	354				
Tumor free	202	Reference			
With tumor	153	2.317 (1.590-3.376)	**<0.001**	1.773 (1.183-2.657)	0.006
Gender	373				
Male	253	Reference			
Female	121	1.261 (0.885-1.796)	0.200		
Age	373				
<=60	177	Reference			
>60	196	1.205 (0.850-1.708)	0.295		
Histologic grade	368				
G1&G2	233	Reference			
G4&G3	136	1.091 (0.761-1.564)	0.636		
AFP(ng/ml)	279				
<=400	215	Reference			
>400	65	1.075 (0.658-1.759)	0.772		
Vascular invasion	317				
No	208	Reference			
Yes	110	1.344 (0.887-2.035)	0.163		
ZCCHC17	373	1.909 (1.442-2.526)	<0.001	1.723 (1.269-2.339)	<0.001

The bold values represent different pathological features. The P value in bold indicates that the P value is statistically significant.

### Functional Analysis of ZCCHC17

**Figure 5:**
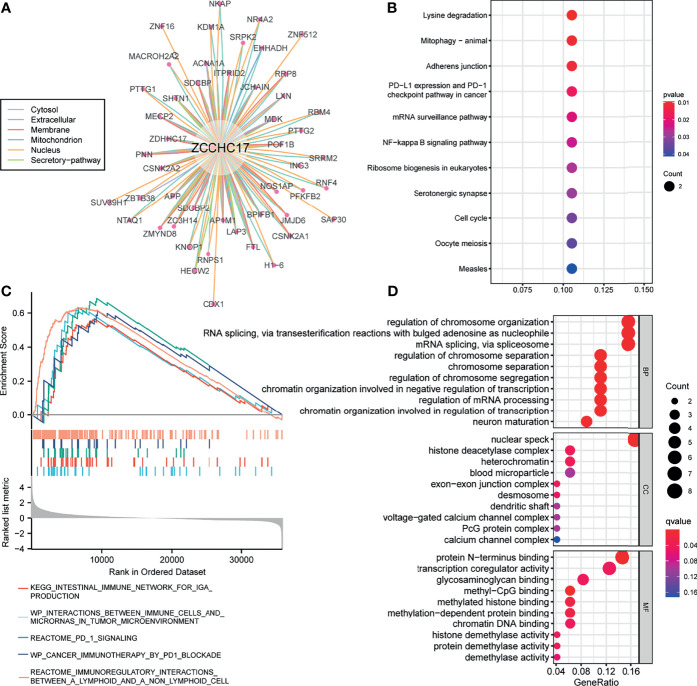
Analysis of function and pathways for ZCCHC17. **(A)** The protein-protein interaction network of ZCCHC17; **(B)** KEGG pathway analysis for proteins interacted with ZCCHC17; **(C)** GSEA analysis for ZCCHC17 in TCGA; **(D)** GO analysis for proteins interacted with ZCCHC17.

Figure A shows 49 proteins that interact with ZCCHC17. The number of lines between the two proteins indicates the number of evidence of the interaction between the two proteins. The lines with different colors represent the positions of the interactions between the two proteins in other evidence (**Figure 5A**). Then we performed KEGG and GO enrichment analysis on these 49 interacting proteins (**Figures 5B, D**). The analysis results show that these genes are mainly related to methylation and participate in immunotherapy-related pathways. Concurrently, we also performed GSEA enrichment analysis on ZCCHC17 in the TCGA dataset, and the results showed that interactions between immune cells and microRNAs in the tumor microenvironment, the intestinal immune network for IgA production, PD-1 signaling, cancer immunotherapy by PD-1 blockade, immunoregulatory interactions between a lymphoid and a non-lymphoid cell (**Figure 5C**). It shows that the highly expressed ZCCHC17 significantly participates in multiple immunotherapy-related pathways and the vital role of ZCCHC17 in the body’s immunity.

### The Correlation Between ZCCHC17 and Immune Cells Infiltration in HCC

**Figure 6:**
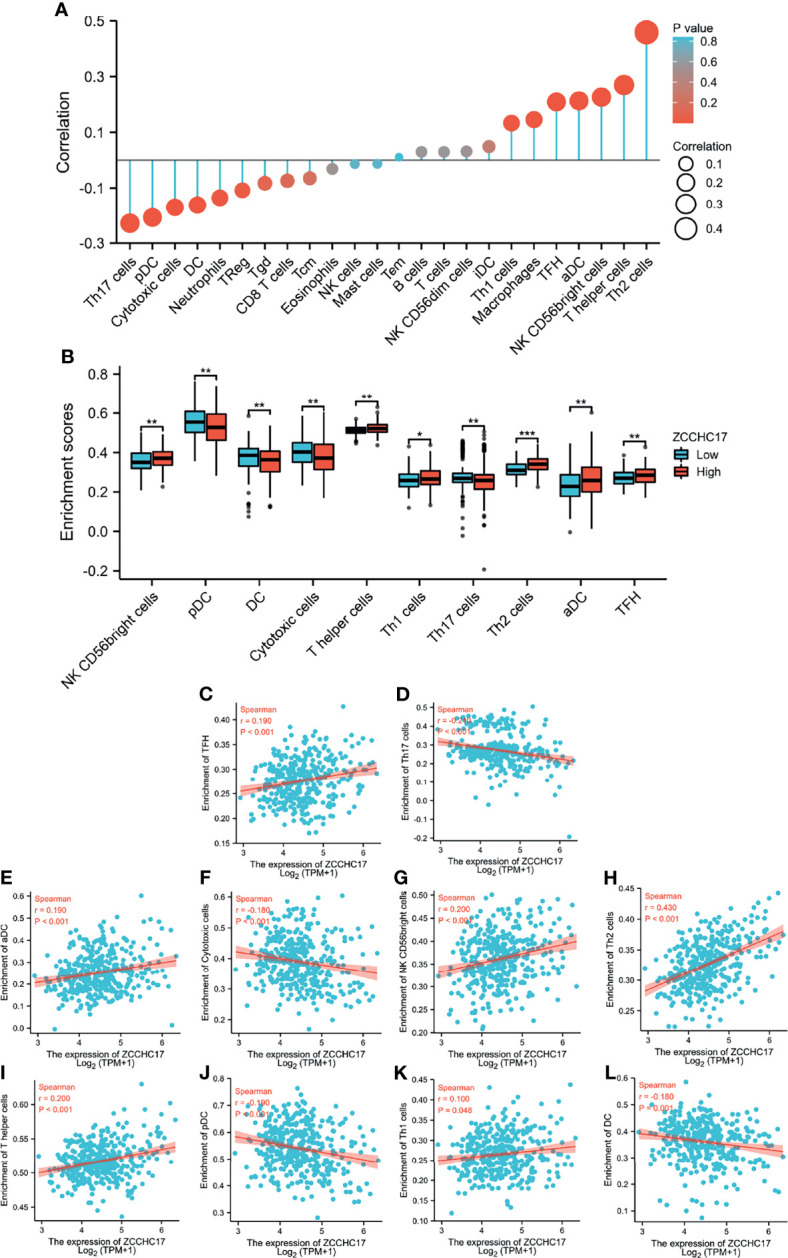
The relationship between ZCCHC17 expression and immune cells infiltration in TCGA. **(A)** Lollipop diagram showing a correlation between ZCCHC17 and levels of 24 immune cell infiltrates; **(B)** 10 immune cells are differentially expressed in different groups of ZCCHC17 (P< 0.05); **(C–L)** Scatter plot showed the correlation between the infiltration of 10 immune cells and the ZCCHC17 expression (P <0.05). *P < 0.05; **P < 0.01; ***P < 0.001.

Based on the previous functional analysis, we found that ZCCHC17 may play an important role in the immune response of HCC patients. Therefore, we used ssGSEA to analyze the infiltration of 24 kinds of immune cells in TME of HCC patients. Spearman method was used to analyze the correlation between the expression of ZCCHC17 and the degree of infiltration of 24 kinds of immune cells. The lollipop graph showed the correlation and significance of the expression level of 24 kinds of immune cells and ZCCHC17 (**Figure 6A**). Further, we analyzed 10 types of immune cells with different infiltration degrees between groups with high and low expression of ZCCHC17 and visualized the correlation of these 10 cells(**Figures 6B–L**). The results showed that the infiltration degree of NK CD56bright cells (r=0.200, P<0.001), TFH (r=0.190, P<0.001), aDCs (r=0.190, P<0.001), Th1 cells (r=0.100, P=0.048), T helper cells (r=0.200, P<0.001) and Th2 cells (r=0.430, P<0.001) in the high ZCCHC17 expression group was significantly higher than that in the low ZCCHC17 expression group, and the infiltration degree of these immune cells was positively correlated with the expression level of ZCCHC17; The infiltration degree of Cytotoxic cells (r=0.180, P<0.001), Th17 cells (r=0.210, P<0.001), pDC (r=0.190, P<0.001) and DC (r=-0.180, P=0.001) in the high ZCCHC17 expression group was significantly lower than that in the low expression group, and the infiltration degree of these immune cells was negatively correlated with the expression level of ZCCHC17.These results suggest that ZCCHC17 may be involved in regulating the TME in HCC. Ns, P≥0.05; *, P<0.05; **, P<0.01; ***, P<0.001

### Response to Immunotherapy in HCC Was Associated With ZCCHC17 Expression Levels

**Figure 7:**
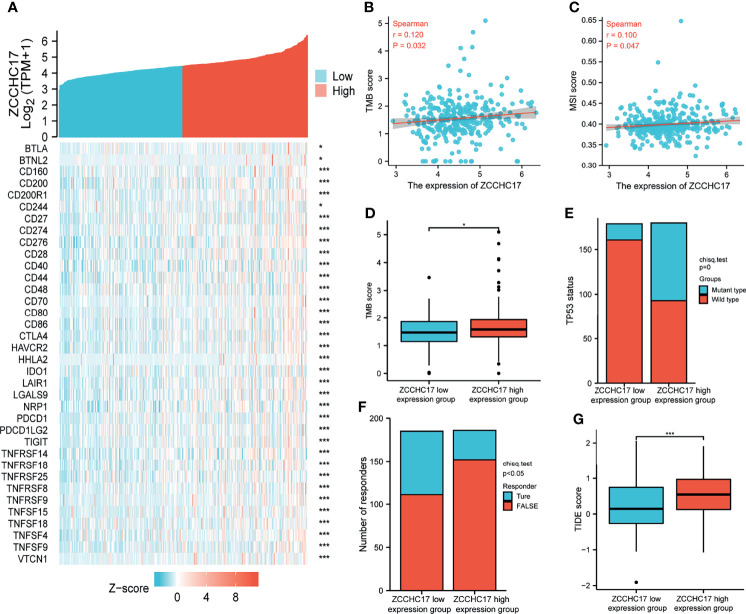
Analysis of ZCCHC17 was used as a marker for immunotherapy. **(A)** Spearman correlation analysis of ZCCHC17 expression and immune checkpoint genes expression; **(B)** Spearman correlation analysis of TMB and ZCCH17 gene expression (r=0.120, P =0.032); **(C)** Spearman correlation analysis of MSI and ZCCH17 gene expression (r=0.100, P =0.047); **(D)** Difference analysis of TMB in different groups of samples of HCC patients (P <0.05); **(E)** Distribution of TP53 gene mutation status in HCC patients in different groups of samples (P <0.001); **(F)** The distribution of immune responses of samples in different groups in the prediction results (P <0.05); **(G)** The distribution of immune response scores in different groups in the prediction results (P <0.001). *P < 0.05; ***P < 0.001.

The previous sections suggest that ZCCHC17 may participate in the body’s immune system and play an important role. Therefore, we collected more than forty common immune checkpoint genes and analyzed the expression correlation between ZCCHC17 and these genes. The analysis results show that ZCCHC17 is positively correlated with the expression of multiple immune checkpoint genes (**Figure 7A**). In addition, the expression level of ZCCHC17 is also significantly positively correlated with the TMB and MSI of HCC patients. That is, the higher expression of ZCCHC17, the higher TMB and MSI for HCC patients (**Figures 7B, C**). The TMB of the ZCCHC17 high expression group was also significantly higher than that of the low expression group (**Figure 7D**); the results of the chi-square test showed that the proportion of patients with TP53 mutation in HCC patients in the ZCCHC17 high expression group was also significantly higher than that of the ZCCHC17 low expression group (**Figure 7E**). Simultaneously, we also use TIDE to predict the results of immunotherapy analysis. TIDE uses a set of gene expression markers to evaluate two different tumor immune escape mechanisms, including the dysfunction of tumor-infiltrating cytotoxic T lymphocytes (CTL) and the rejection of CTL by immunosuppressive factors. A high TIDE score indicates poor immune checkpoint blockade(ICB) treatment and prognosis after ICB treatment. The results showed that the proportion of patients in the ZCCHC17 high expression group that responded to immunotherapy was significantly lower than that of the ZCCHC17 low expression group (**Figure 7F**); The TIDE score of the ZCCHC17 high expression group was significantly higher than that of the low expression group (**Figure 7G**). Collectively, these analyses suggest that HCC patients with ZCCHC17 high expression had worse therapeutic effects than those with ZCCHC17 low expression during immunotherapy. Ns, P≥0.05; *, P<0.05; **, P<0.01; ***, P<0.001

## Discussion

ZCCHC17, so far, is still a relatively unresearched human protein-coding gene. Previous research reports indicate that ZCCHC17 is involved in a series of biological processes, including transcription regulation (16) and regulation of mRNA splicing (17), suggesting that ZCCHC17 may act as a transcriptional cofactor and/or play a role in regulating mRNA splicing. Another study showed that it is the primary regulator of synaptic gene expression in Alzheimer’s disease (5). However, its clinical significance in tumors and its role and function have not yet been elucidated. The incidence of HCC in China accounts for 55% of the world’s HCC cases. It is urgent to find effective diagnostic and therapeutic targets. Therefore, in this study, we combined clinical specimens and public databases from TCGA, ICGC, GEO to analyze the role and significance of ZCCHC17 in HCC. The results show that mRNA and protein expression of ZCCHC17 in HCC tissues is higher than normal liver tissues. This abnormally high expression is related to the abnormal methylation of ZCCHC17. Subsequently, further analysis results showed that the high expression of ZCCHC17 was significantly related to multiple tumor malignant indicators. In addition, the survival rate of HCC patients with high ZCCHC17 expression was significantly lower than that of HCC patients with low expression. Finally, we further studied the function of ZCCHC17 and its potential role in clinical treatment. We found that it plays a vital role in the immune system, regulates immune cells in TME, and may serve as an important marker for immunotherapy. All in all, these results confirm that ZCCHC17 is used as an HCC diagnosis and as an independent prognostic biomarker and may promote the development of tumor-targeted therapy and tumor immunotherapy. The hypothetical mechanism and the role of ZCCHC17 in HCC are shown in **Supplementary Figure 1**.

Unlike the routine detection of HER2, EGFR, BRAF, KRAS and other genes in breast cancer, lung cancer, colorectal cancer and other tumors, HCC has not yet an apparent gene-phenotype related to its prognosis and treatment due to its heterogeneity (18). Therefore, genetic testing is not recommended for routine clinical use in diagnosing HCC, according to the 8th edition of the American Joint Committee on Cancer. AFP (19) is more sensitive in HCC (especially hepatitis B-related HCC), but it must be combined with imaging because it is a non-specific tumor marker. For this reason, this study is the first to analyze the role of ZCCHC17 as a tumor diagnostic marker. First, ZCCHC17 in tumors is significantly higher in mRNA and protein levels than in normal adjacent tissues. We found that ZCCHC17 has strong specificity and can effectively distinguish tumor and non-tumor samples. Therefore, we did a methylation analysis to explore further the reasons for the abnormally high expression of ZCCHC17 in tumor samples. DNA methylation is an extensively researched epigenetic modification in regulating gene expression (20). Although the nucleotide sequence and composition of DNA are not changed after DNA methylation, gene expression is affected. By downloading the methylation data of HCC patients from TCGA and analyzing the correlation between the methylation level of different CpG sites and the expression level of ZCCHC17, we found that the methylation level of ZCCHC17 in normal tissues was significantly higher than that in tumor tissues. This also explains the reason for the abnormal expression of ZCCHC17 in HCC tissues to some extent.

Because of the abnormal expression of ZCCHC17 in HCC, we analyzed its clinical significance in HCC patients. On the one hand, the significant difference between ZCCHC17 and AFP (ng/ml) at different levels emphasizes the feasibility of ZCCHC17 as a diagnostic marker, which is consistent with the results of our previous analysis. On the other hand, ZCCHC17 has significant differences in different groups’ histologic grades, tumor status, vascular invasion, and pathological stage. Meanwhile, we also noticed that ZCCHC17 is always expressed higher in higher histological grades, higher pathological stages, and tissues with vascular invasion. It is well-known that high-grade tumors are poorly differentiated, and grow very fast and are prone to distant metastasis. These results indicate that ZCCHC17 is also helpful for evaluating the malignant degree of tumors. At the same time, it suggests that ZCCHC17 may promote the malignant transformation of tumors, but its specific mechanism also leads us to study further. Secondly, we use the KM-survival curve and Cox regression analysis to analyze the expression of ZCCHC17 on the prognosis of patients. Of note, the results also showed significant relationships between ZCCHC17 and the prognosis of HCC patients in the analysis of different cohorts.

In addition, for the methylation levels of different methylation sites, we also analyzed the relationship between the methylation levels of different methylation sites and the prognosis in HCC patients. The results showed that the methylation level of multiple methylation sites in ZCCHC17 was related to the prognosis of HCC patients. More than ten years ago, biomarkers based on DNA methylation were considered the next “big event” in cancer biomarker research, but so far, they have been unable to meet expectations. In addition to this, the high cost of next-generation sequencing technologies also makes it difficult for this marker to be further promoted. In addition, the complex relationship between DNA methylation and its precise genomic location is one of the main obstacles. Like the results in this study, the level of methylation at different sites has very different effects on the prognosis of patients. In recent years, studies have shown that methylation of the RIZ1 gene promoter region in HCC tissues is associated with postoperative tumor recurrence, which may be the reason for the poor prognosis of patients with RIZ1 gene methylation positive in HCC tissues (21); Similarly, another study also showed that Compared with normal tissues, the methylation level of the ADRA1A promoter region in the tumor tissues of HCC patients was significantly increased, the mRNA and protein levels of ADRA1A were decreased, and DNA methyltransferase inhibitors could increase the mRNA expression of ADRA1A (22). It shows that ADRA1A gene hypermethylation may be involved in the occurrence of HCC. These studies have shown that DNA methylation is closely related to the progress of HCC. Nevertheless, the downside is that they all only paid attention to the impact of DNA methylation but failed to clarify the methylation sites that affect the prognosis of HCC patients in detail. This study used high-throughput sequencing data to more accurately determine the methylation sites of ZCCHC17 that affect the prognosis of patients during the development of HCC, indicating that abnormal gene methylation is also a fundamental reason for the prognosis of HCC patients. To a certain extent, this result also provides further valuable clues for the precise treatment and prognosis evaluation of HCC.

It is good to understand the complete function by analyzing its specific and mutual binding proteins and probing the signal pathway and function involved in a protein. Therefore, we analyzed the proteins that interacted with ZCCHC17. Through the enrichment analysis of KEGG and GSEA pathways, we noticed that ZCCHC17 is mainly involved in the body’s immune system in terms of mechanism regulation and regulating a series of immunotherapy-related processes, including cancer immunotherapy by PD-1 blockade, PD-L1 expression and PD-1 checkpoint pathway in cancer, these analyses emphasize the link between ZCCHC17 and immune response. In the GO enrichment analysis results, ZCCHC17 and its related proteins are mainly involved in the body’s methylation process, which is consistent with the results of our previous analysis and proves the reliability of this study.

More and more research has focused on immune cells’ crucial role in the TME. Under normal circumstances, the immune system can recognize and eliminate tumor cells in the TME. However, tumor cells can adopt different strategies to suppress the human immune system to survive and grow. Our analysis results show that ZCCHC17 is significantly related to the infiltration of various immune cells. It was previously reported that regulatory T cells (Treg) with CD4^+^ and CD25^+^ are the most significant immunosuppressive cell groups in the tumor microenvironment. Their existence has prognostic significance because the accumulation of Treg is related to poor prognosis and aggressiveness of HCC (23). At the same time, we noticed the degree of correlation between ZCCHC17 and Th1 cells and Th2 cells. Th cells secrete cytokines, which can regulate the body’s immune function. Th cells are divided into Th1 and Th2. Th1 cells secrete cytokines IFN-γ, TNF-α, IL-12, which play an essential role in tumor immunity (24), and IL-4, IL-6, and IL-10 are cytokines secreted by Th2 cells, which can inhibit the secretion of cytokines by Th1 cells and promote humoral immunity (25). Th1/Th2 balance plays a vital role in the maintenance of normal immune function. Under normal circumstances, Th1 cell function is relatively strong. Th2 cell function is hyperactive and then synthesizes and secretes many inhibitory cytokines under an imbalance of Th1/Th2 condition, which directly leads to the decline of the body’s immune function and causes the immune escape of tumor cells (26). Our research has shown that ZCCHC17 is highly correlated with immune cells in TME, indicating that ZCCHC17 may help tumor cells to immune escape by affecting the Th1/Th2 balance, thereby promoting the occurrence of HCC. However, its exact mechanism needs to be further confirmed. Nevertheless, no matter what, these studies suggest that we pay attention to the relationship between genes and immune cells to help understand the occurrence and development of tumors and contribute to the development of immunotherapy therapies.

Based on the functional analysis of ZCHHC17 and its high correlation with the degree of immune cells infiltration in TME, it is suggested that it plays a vital role in regulating immune mechanism function. Therefore, here we have collected more than forty common immune checkpoint genes, including monoclonal antibody immune checkpoint inhibitors, therapeutic antibodies, cancer vaccines and small molecule inhibitors. We analyzed the correlation between the expression of ZCCHC17 and these immune checkpoint genes, and the results showed that ZCCHC17 is positively correlated with the expression of multiple immune checkpoints. Immunotherapy is a treatment method that restores the body’s normal anti-tumor immune response by restarting and maintaining the tumor-immune cycle, thereby controlling and eliminating tumors. Nevertheless, it is difficult to find those patients who can benefit from immunotherapy. Most previous studies believe that MSI, TMB and ICB-related gene expression is closely related to response to immunotherapy (27, 28). However, they cannot independently accurately predict the treatment effect. Therefore, we further analyzed the relationship between ZCCHC17 and TMB, MSI and other immunotherapy markers, and the analysis results also showed a significant correlation between ZCCHC17 and these markers. Therefore, we infer that the expression of ZCCH17 can be used as a marker of the immunotherapy effect of patients. We further applied TIDE to predict the immunotherapy effect of HCC patients in different ZCCHC17 expression groups. Intriguingly, the results of TIDE showed that patients in the ZCCHC17 high expression group showed a worse response to the immunotherapy. In contrast, patients in the ZCCHC17 low expression group obtained better therapeutic benefits from immunotherapy.

With the development of immunotherapy, many corresponding clinical studies have been carried out. Although specific results have been achieved, the situation is still not satisfactory. A recent study shows that the idea of ​​immunotherapy for solid tumors with a high tumor mutation burden(TMB-H) is based on tumor mutations that may produce immunogenic neoantigens. The increase in neoantigens is positively correlated with the increase in the count of CD8^+^ T cells, and CD8^+^ T cells infiltrated tumors are the basis for a good response to immunotherapy (29). Therefore, TMB-H may not be suitable as a biomarker for predicting the efficacy of immunotherapy for all cancers. Cervical squamous cell carcinoma and adenocarcinoma, endometrial cancer, melanoma, lung cancer and bladder cancer are defined as type-I cancers (30). TMB-H can be used as a predictor of immunotherapy efficacy for type-I cancers. However, HCC, pancreatic cancer, esophageal cancer, and other tumors are considered type-II cancers. The evidence for TMB-H to predict the efficacy of immunotherapy for type-II cancers is still insufficient (30). The predictive value of TMB in another study from another study is still only reflected in NSCLC, head and neck squamous cell carcinoma and melanoma. However, the predictive value of TMB-H is not observed for other cancer types (31). These conclusions also put forward the limitations of the current immunotherapy effect prediction to some extent. It indicates that the prediction of the effects of immunotherapy may require more markers or target combined analysis to predict effectively.

This study has improved our understanding of the relationship between ZCCHC17 and the development of HCC, but this study still has some limitations. First, although we analyzed different cohorts, we verified the differential expression and clinical significance of ZCCHC17 in HCC from the level of mRNA and protein. However, this study has not proven its direct mechanism of action in HCC, but this is also one of the directions we are currently working on. In addition, concerning evaluating the effects of immunotherapy in patients with HCC, although we have used the tools currently recognized as the best evaluation of the effects of immunotherapy, we have reached a different conclusion from some previous studies. However, because we are currently unable to obtain a large-scale immunotherapy cohort to verify the prediction of immunotherapy effects in this study, we conservatively propose that the evidence for TMB or MSI as a marker of immunotherapy for HCC patients is insufficient, and hope that in the future, we can collect more information to validate our conclusions.

All in all, this study started from ZCCHC17, explored the role of ZCCHC17 in HCC and clarified its clinical significance in more detail. The collection of bioinformatics and clinical samples proved that ZCCHC17 is highly expressed in HCC tissues, and this high expression is related to the poor prognosis of patients. At the same time, this study found that ZCCHC17 is involved in immune-related pathways in HCC patients and is also related to multiple immunotherapy-related targets (immune checkpoint genes, TMB, MSI). These findings provide a new and effective molecular marker for the diagnosis and targeted therapy of HCC. It also provides some new insights for judging the prognosis of HCC patients and evaluating the effects of immunotherapy. These findings can promote our current understanding of the role of ZCCHC17 in HCC and promote its transformational application in HCC diagnosis and treatment.

## Data Availability Statement

The datasets presented in this study can be found in online repositories. The names of the repository/repositories and accession number(s) can be found in the article/**Supplementary Material**.

## Ethics Statement

The studies involving human participants were reviewed and approved by Affiliated Hospital of Youjiang Medical University for Nationalities The Ethics Committee. The patients/participants provided their written informed consent to participate in this study.

## Author Contributions

FL and JL collected the data and wrote the manuscript. PL, LZ and WH participated in data analysis and clinical sample collection. CL and XW designed the study. All authors contributed to the article and approved the submitted version.

## Funding

This study was supported by National Natural Science Foundation of China (Nos. 81341122, 81970462), Natural Science Foundation of Guangxi Zhuang Autonomous Region (Nos. 2020GXNSFAA259052), High-level Talents Project of Affiliated Hospital of Youjiang Medical College for Nationalities (R202011708,Y20196301).

## Conflict of Interest

The authors declare that the research was conducted in the absence of any commercial or financial relationships that could be construed as a potential conflict of interest.

## Publisher’s Note

All claims expressed in this article are solely those of the authors and do not necessarily represent those of their affiliated organizations, or those of the publisher, the editors and the reviewers. Any product that may be evaluated in this article, or claim that may be made by its manufacturer, is not guaranteed or endorsed by the publisher.

## Supplementary Material

Supplementary Figure 1 The hypothetical mechanism and the role of ZCCHC17 in HCC.

Supplementary Figure 2 The correlation between ZCCHC17 and the markers of regulatory T cells in HCC.
