# Supplementary figures and images for: ZCCHC17 Served as a Predictive Biomarker for Prognosis and Immunotherapy in Hepatocellular Carcinoma
Source: Front Oncol. 2022 Jan 6;11:799566. doi: 10.3389/fonc.2021.799566 (PMC8770814; doi:10.3389/fonc.2021.799566)

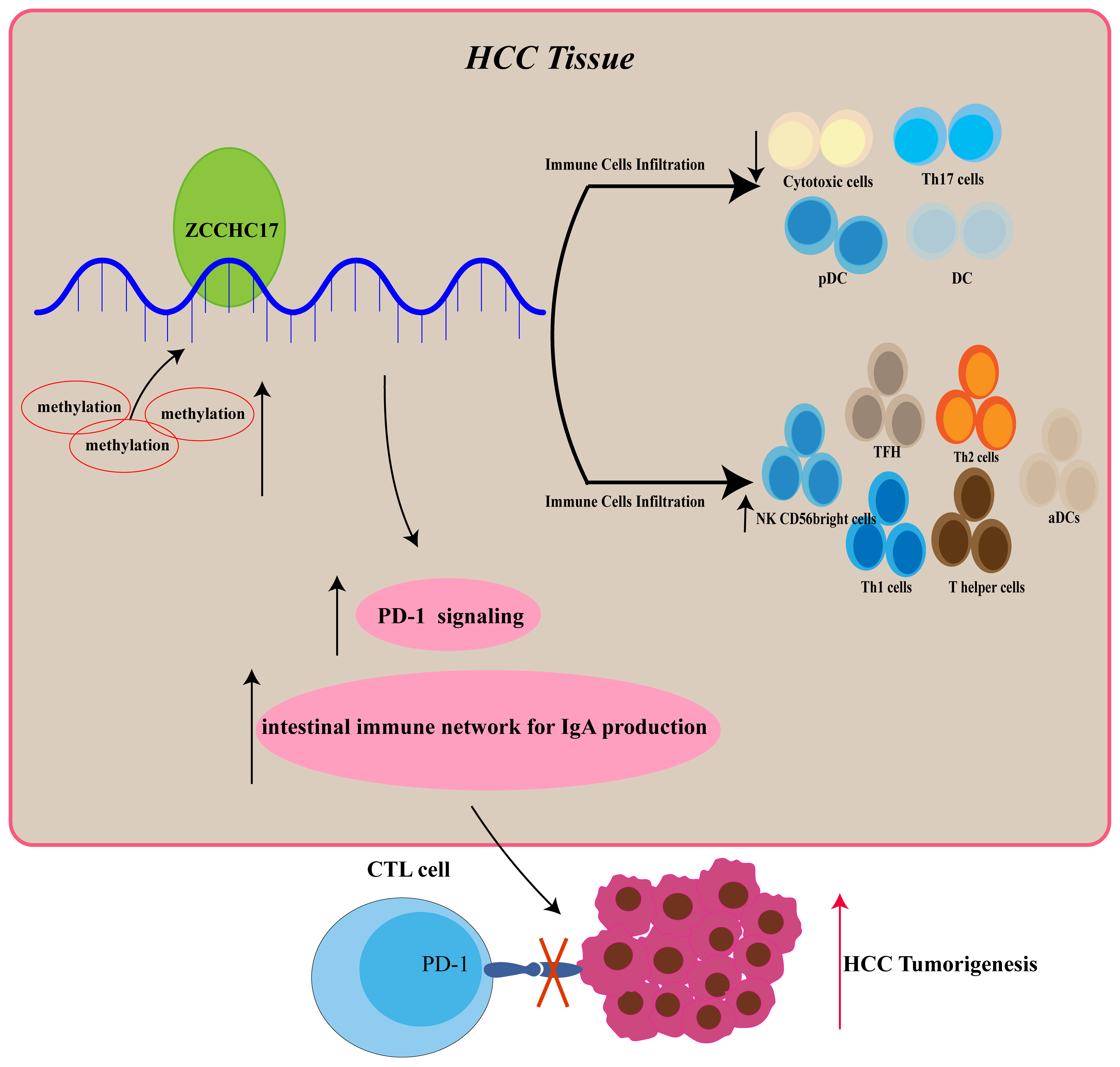

Supplement: Supplementary Figure 1 — The hypothetical mechanism and the role of ZCCHC17 in HCC. [file Image_1.tif]

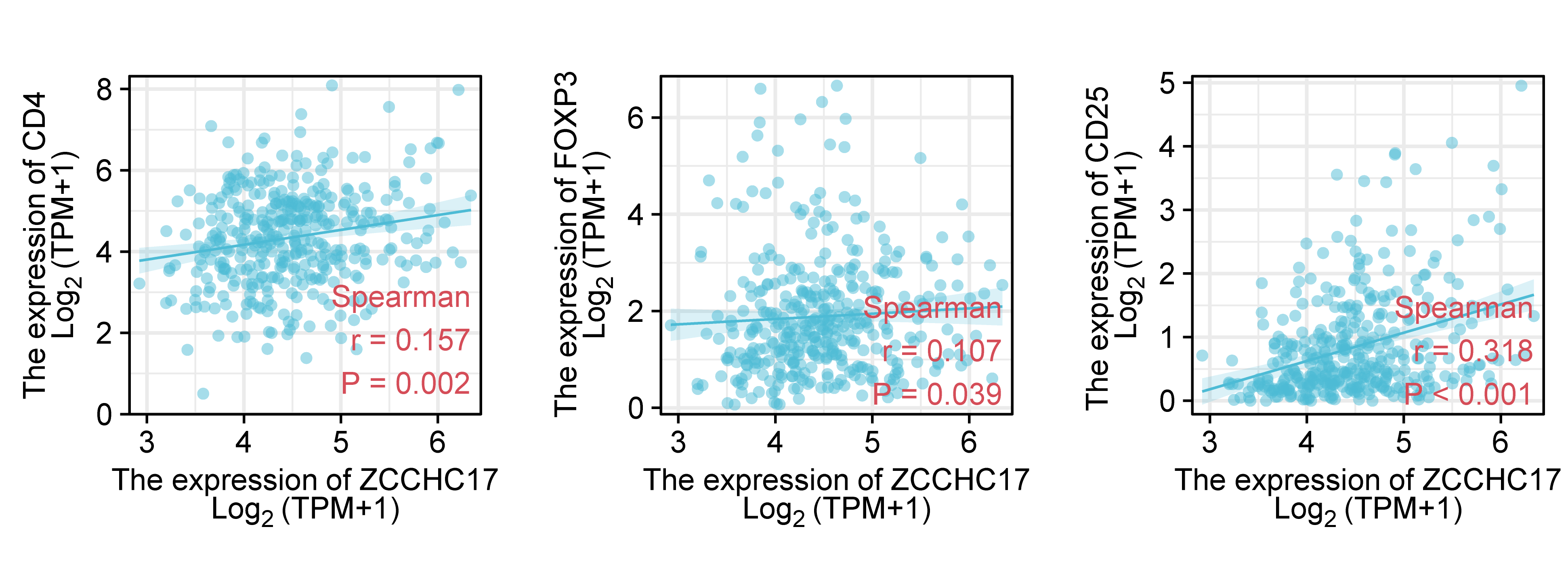

Supplement: Supplementary Figure 2 — The correlation between ZCCHC17 and the markers of regulatory T cells in HCC. [file Image_2.tif]
